# Items of Medical Interest in New York

**Published:** 1892-03

**Authors:** Wm. L. Russell

**Affiliations:** 151 East 50th St. New York


### ITEMS OF MEDICAL INTEREST IN NEW YORK.

NEW YORK, February 15, 1892.

### TUBERCULOSIS.

The interest aroused in everything pertaining to tuberculosis by the introduction of Koch's supposed remedy has continued with slight abatement here during the present winter. It is now almost generally admitted that the disease is contagious, and that it is not unfrequently propagated by communication. This aspect of the subject was considered by Dr. Janeway, in a paper read before the Academy, not long since. He stated that he believed heredity to be a prominent factor in causation, and that the germ introduced during fœtal or infantile life might spring into activity in later years. Some cases, however, unquestionably originated through contagion. He had seen a number of instances of this; e. g, where all the children of a family in which there was no hereditary taint had died one after the other until the parents only were left, no precautions having been taken to avoid contagion; where a post-office clerk of healthy parentage had become affected through working in the same room with a fellow clerk who was a victim of phthisis; where an infant had contracted the disease by being kissed by a patient in whose lungs cavities existed.

A question of great pratical importance related to the position occupied by the meat and milk of animals in the production of the disease in human beings. The concentration of people in cities and large towns, which seemed to be the tendency of modern times, was to be regarded with grave apprehension in relation to the spread of tuberculosis and other contagious diseases. The further concentration in large flats and apartment houses was a still greater menace to the public health. The results of this over-crowding might be looked forward to with apprehension.

If then the contagious nature of tuberculosis were admitted, it became our duty as medical men to inculcate the necessity of destroying all tuberculous discharges. Phthisis patients should be under the absolute control of the medical profession ; a report should be sent to some central authority from whom printed regulations should emanate. There should also be inspectors to visit large apartment houses, and exercise a supervision over the methods of prevention employed, giving instruction and advice wherever occasion arose. Destruction of sputa, cleansing of the mouth, bedding, and person of tuberculous patients, frequent washing and disinfection of the floor and walls of the rooms occupied by such patients, and the protection of parts not frequently cleansed were some of the points to be attended to. The well should not occupy rooms with the sick, and the intermarriage of well and sick should be discouraged. At the hotels at health resorts, rooms which had been occupied by phthisis patients should not be carelessly passed over to a healthy new arrival. They should first be carefully cleaned and ventilated, and certain rooms should be arranged so as to render these processes easy of accomplishment. Cuspidores should always contain some fluid, and should be disinfected regularly with boiling water. It was surely not too much to expect, too, that bodies of people working together in rooms should be periodically inspected so as to prevent the introduction of the elements of contagion.

Dr. John S. Billings, of the United States Army, said, in continuing the discussion, that he had estimated that there were about 125,000 consumptives in the United States. The influence of the aggregation of people into cities was very noticeable as a factor in causation. He believed that the dry and pulverized sputa of phthisis patients was the chief cause of the disease. The crowding, uncleanliness, and debilitating habits of the criminal and vicious classes rendered them less resistive, and thus more liable to the development of phthisis ; a criminal court-room was a particularly dangerous place.

Dr. Jacobi was of the opinion that in considering the prevention of tuberculosis in children, it was safe to assume in most cases that the disease was introduced from without. The avenues of introduction were the skin and mucous membranes. If these were sound the entrance of germs of any kind was difficult. Diphtheria did not develop on a healthy mucous membrane, and what was true of diphtheria was probably equally true of other communicable diseases. An eczema, or a naso-pharyngeal catarrh should not therefore be regarded as unimportant; they should be cured. The same was true of diarrhœa. Lymphatic glands became enlarged frequently, as a result of mucous catarrhs, and there the bacilli of tuberculosis might find lodgement, to be eventually conveyed to other tissues. Such enlarged glands should be enucleated if possible. The possibility of contagion from milk was also to be thought of, and the simple precaution of boiling should be adopted to render it harmless.

Dr. J. W. Roosevelt thought that we might go too far in these matters, and that it would be better to restrict our efforts to raising the standard of cleanliness, and to the destruction of sputa.

Dr. W. H. Porter would feed our infants so as to insure the possession of soil in which tuberculosis could not secure a footing.

Dr. C. G. Currier had reviewed the current literature on the subject, and had found that heredity was still regarded as a prominent factor in causation.

Dr. Lawrence Johnson believed that great injury would be inflicted on the sick by removing the element of hope which often sprang from ignorance of the nature of the disease. Our efforts should be directed to inculcating the necessity of more careful cleanliness.

Dr. A. Seibert would discourage the habit of kissing, so common among school girls.

The President of the Academy, Dr. Loomis, said that while it had been demonstrated that tuberculosis was to be classed among contagious diseases, some care was necessary in announcing the fact to the general public. If it were understood that the disease was contagious in the general sense, a panic might result, and the victims would be shunned as lepers were in olden times. A process of gradual education was necessary, and in the meantime the importance of general cleanliness should be urged, with the abandonment of the disgusting and unnecessary habit of spitting, so prevalent in this country. The value of fresh air and exercise had not been sufficiently appreciated in the past, but we were improving in this respect. * * *

The subject was still further discussed at the last meeting of the Academy. Dr. Roosevelt read a paper, in which he said that to wage an effective war against the bacillus of tuberculosis we should have to segregate all who were known to be diseased, and all "suspects." All diseased animals would have to be killed, and the most of our food sterilizied. Infected houses would have to be burned. Even with such extreme measures, two or three generations would have to pass before any marked results would be noticed. Such a plan of procedure was of course absurd, and he believed that the free use of soap and water and fresh air were rather to be thought of than sulphur fumigation and other "heathen rites." The internal administration of germicides was also irrational.

Dr. C. E. Quimby said that though we might admit that there was no phthisis without tuberculosis, and no tuberculosis without the bacillus, it would not do to forget that back of all was the lung. The war of extermination against the bacillus was hopeless; would it not, then, be better to direct our efforts toward improving the qualities of lungs, that they might set it at defiance? This was possible, he believed, by improved nutrition. Alcoholism was a most prominent cause of malnutrition, and failure to live wholesome physical and moral lives.

Dr. F. W. Jackson thought that sentiment should not enter into the consideration of the subject, nor should we question whether this or some later generation should see the fruits of our labors. If we were satisfied concerning the contagious nature of phthisis, we should tell our patients so, and advise methods of prevention. The introduction of the bacilli by means of milk and meat from diseased animals should also be prevented, and precautions should be taken against allowing the well to breathe the expired air of the sick; they should not be allowed to occupy the same bed. He believed in disinfecting sputa, and spoke favorably of the rules issued by the Board of Health.

DIPHTHERIA.

This important subject was again under consideration in the Section on Pædiatrics at the last meeting. Dr. J. E. Withers introduced the subject by stating his views in reference to the best disinfectant and apparatus for use in treating the throat and nose. Cleanliness and destruction of such germs as could be reached were the points to aim at. Any substance that irritated the mucous membrane, or method of treatment which exhausted the child, was injurious. For treatment of the nose, irrigation by means of a Davidson's syringe was the most thorough and satisfactory method to employ. A ten per cent. solution of peroxide of hydrogen, or a saturated solution of boracic acid, was the fluid used by him. This, forced into one nostril, would flow from the other and from the mouth, and thus come in contact with the whole seat of disease. The irrigation should be repeated every two hours, or if there was much obstruction, discharge or fetor, every hour. The inhalation of sulphurous acid gas, developed by burning a sulphur candle in the sick room, was of some service. He had experienced in his own person, when suffering from diphtheria, more relief from the dryness and soreness of the throat after such an inhalation than from any other measure employed. Observation had also shown that the redness and swelling decreased under its use. The inhalation of medicated steam promoted the separation of the membrane. A croup kettle, with a piece of flexible rubber tubing attached, was the proper apparatus, care being taken to have the tube long enough to allow the steam to be brought into close contact with the child. To syringe out the pharynx, a piece of soft rubber tubing attached to the Davidson could be passed along the cheek, back of the last molar, and so to the seat of disease.

Dr. H. D. Chapin spoke on the methods of quarantining. He advocated the establishment of stations or houses by the city authorities to which exposed well children could be sent when proper means for their protection could not be provided at home.

Dr. L. Emmet Holt considered feeding and methods of forced feeding. He spoke strongly against the mistake of over-feeding or stimulating. He did not believe in ice-cream or wine-jellies as suitable articles of diet, however easily they might be swallowed. Diluted milk, hot or cold as preferred, with a little cocoa added if necessary, mutton broth, junket, and koumiss were the articles to be relied upon for young children. Nothing was better than brandy, as a stimulant, but whatever the child could most easily be induced to take, was best for that case. The stimulant might be diluted with mineral water, but was not to be mixed with the food. Food should be given at intervals of two hours, and in smaller quantities than in health ; stimulants might be given more frequently. The latter should only be given to meet indications, and should then be made to produce the desired effect. Small quantities frequently produced the best and quickest results. Forced feeding was in some cases demanded by the fifth to the tenth day, when the child began to refuse all food and stimulants. The rectum was sometimes resorted to under such circumstances, but it seldom retained more than one or two injections. It was his custom to feed through a tube passed well into the œsophagus, through the mouth if possible, or where there was great resistance, through the nose. Food and stimulants could be given together in this way, at intervals of four hours. Peptonized milk was the best form of food to employ.

Dr. Jacobi said in relation to the constitutional treatment, that many had come to rely entirely on bichloride of mercury. This could be safely given in doses of one-quarter to one-half a grain in twenty-four hours to children as young as four or five months of age. Numerous small doses well diluted in eight or ten thousand parts of water was the best mode of administration, The inhalation of fumes from evaporating calomel was being tried at the Foundling Asylum, it was thought with some benefit. Ten grains of calomel were evaporated for ten minutes under a tent which was allowed to remain over the child five minutes longer. This was repeated every three hours.

WM. L. RUSSELL.

151 *East* 50*th St.*

